# Supplementary material for: Immunological synapse formation between T regulatory cells and cancer-associated fibroblasts promotes tumour development
Source: Nat Commun. 2024 Jun 11;15:4988. doi: 10.1038/s41467-024-49282-1 (PMC11167033; doi:10.1038/s41467-024-49282-1)
Supplement: Supplementary file 3 — Reporting Summary [file 41467_2024_49282_MOESM3_ESM.pdf]

nature portfolio

Corresponding author(s): Dr. Panayotis Verginis

Last updated by author(s): Mar 19, 2024

Reporting Summary

Nature Portfolio wishes to improve the reproducibility of the work that we publish. This form provides structure for consistency and transparency in reporting. For further information on Nature Portfolio policies, see our Editorial Policies and the Editorial Policy Checklist.

Statistics

For all statistical analyses, confirm that the following items are present in the figure legend, table legend, main text, or Methods section.

n/a	Confirmed	
☐	☒	The exact sample size (*n*) for each experimental group/condition, given as a discrete number and unit of measurement
☐	☒	A statement on whether measurements were taken from distinct samples or whether the same sample was measured repeatedly
☐	☒	The statistical test(s) used AND whether they are one- or two-sided<br>*Only common tests should be described solely by name; describe more complex techniques in the Methods section.*
☒	☐	A description of all covariates tested
☐	☒	A description of any assumptions or corrections, such as tests of normality and adjustment for multiple comparisons
☐	☒	A full description of the statistical parameters including central tendency (e.g. means) or other basic estimates (e.g. regression coefficient) AND variation (e.g. standard deviation) or associated estimates of uncertainty (e.g. confidence intervals)
☐	☒	For null hypothesis testing, the test statistic (e.g. *F*, *t*, *r*) with confidence intervals, effect sizes, degrees of freedom and *P* value noted<br>*Give P values as exact values whenever suitable.*
☒	☐	For Bayesian analysis, information on the choice of priors and Markov chain Monte Carlo settings
☒	☐	For hierarchical and complex designs, identification of the appropriate level for tests and full reporting of outcomes
☒	☐	Estimates of effect sizes (e.g. Cohen's *d*, Pearson's *r*), indicating how they were calculated

Our web collection on statistics for biologists contains articles on many of the points above.

Software and code

Policy information about availability of computer code

Data collection	1. BD FACS Diva SOFTWARE v 6.1.3 for flow cytometry<br>2. Leica Application Suite X (Leica Microsystems) for microscopy imaging<br>3. Leica video-microscope for time-lapse imaging.<br>4. Illumina Nextseq 500 for RNA sequencing<br>5. Electron Microscope JEOL JEM-2100 for transmission electron microscopy<br>6. Dionex Ultimate 3000 nanoRSLC online with a Thermo Q Exactive HF-X Orbitrap mass spectrometer for LS-MS/MS
Data analysis	1. FlowJo version 10 and v10.7.2 for flow cytometry<br>2. GraphPad Prism version 8.2.1 for graphic representation and statistic analysis of numeric data<br>3. ImageJ Fiji version 2.3.0 distribution for imaging analysis

For manuscripts utilizing custom algorithms or software that are central to the research but not yet described in published literature, software must be made available to editors and reviewers. We strongly encourage code deposition in a community repository (e.g. GitHub). See the Nature Portfolio guidelines for submitting code & software for further information.

Data

Policy information about availability of data

All manuscripts must include a data availability statement. This statement should provide the following information, where applicable:

- Accession codes, unique identifiers, or web links for publicly available datasets
- A description of any restrictions on data availability
- For clinical datasets or third party data, please ensure that the statement adheres to our policy

The data that support the findings of this study are available within the article, Supplementary Information and Source Data file. Source data are provided with this paper; additional data are available from the corresponding author upon reasonable request. The mouse NGS raw data are available through the National Institute of Health (NIH) BioProject ID: PRJNA874541 (Link provided: https://dataview.ncbi.nlm.nih.gov/object/PRJNA874541?reviewer=4m6fv13mct416jj0d009g2tqc2). The mass spectrometry proteomics data have been deposited to the ProteomeXchange Consortium via the PRIDE [1] partner repository with the dataset identifiers PXD047024 regarding CAF lysates and PXD047038 regarding CAF secretome.

Research involving human participants, their data, or biological material

Policy information about studies with human participants or human data. See also policy information about sex, gender (identity/presentation), and sexual orientation and race, ethnicity and racism.

Reporting on sex and gender	For the present study, a total of 3 melanoma cases consisting of 2 female and 1 male participants, as well as 15 colorectal carcinoma cases consisting of 4 female and 11 male participants were assessed. Sex and gender were determined based on self-reporting and were not considered in study design. Consent has been obtained for sharing of individual-level data.
Reporting on race, ethnicity, or other socially relevant groupings	No information was obtained regarding race, ethnicity or other socially relevant groupings.
Population characteristics	A total of 3 melanoma cases from participants aged 40-82 years old were assessed. Diagnosis of the three cases consisted of lentigo malignant melanoma, acral melanoma and superficial spreading melanoma. Furthermore, a total of 8 colorectal carcinoma cases from participants aged 40-82 years old were assessed, while age was not noted in 7 colorectal carcinoma cases which were assessed. 5 CRC participants were classified as TNM stage 2, 9 participants as TNM stage 3 and 1 participant as TNM stage 4.
Recruitment	All cases assessed were from an unbiased selection of patients from which ethical approval was obtained since 2010 (see "ethics oversight" for protocol #).
Ethics oversight	The study was approved by the ethics committee/institutional review board of Attikon University Hospital, Haidari, Athens, Greece (protocol # 2nd Dep. Pathol., EBΔ444/17-12-2010) and conducted in accordance with the 1964 Declaration of Helsinki and its later amendments. Informed consent was obtained to use sociodemographic data from all patients.

Note that full information on the approval of the study protocol must also be provided in the manuscript.

Field-specific reporting

Please select the one below that is the best fit for your research. If you are not sure, read the appropriate sections before making your selection.

☒ Life sciences ☐ Behavioural & social sciences ☐ Ecological, evolutionary & environmental sciences

For a reference copy of the document with all sections, see nature.com/documents/nr-reporting-summary-flat.pdf

Life sciences study design

All studies must disclose on these points even when the disclosure is negative.

Sample size	An estimate of three to nine mice per group were used in individual experiments. This sample size was determined on pilot experiments based on differences between genotypes and treatments and is indicated in the relative figure legends.
Data exclusions	No data were excluded from the analyses.
Replication	Multiple independent experiments were performed and similar results were obtained; experiments which were not performed independently were replicated following a mean time of 3-4 weeks, following completion of analyses and conclusions from previous experiments. Number of replicates per experiment is indicated in the relative figure legends.
Randomization	For experiments comparing mice of the same genotype treated with immunotherapy or analysed in different tumour time-points or inoculated with different cancer cell lines, mice were randomly assigned to the different groups and treatments. For experiments comparing knockout/creflox mice vs. control, groups were set up based on the genotype as indicated in figure legends.

Blinding

The investigators were not blinded to the identities of the samples. Compared samples were collected and analyzed under the same conditions.

Reporting for specific materials, systems and methods

We require information from authors about some types of materials, experimental systems and methods used in many studies. Here, indicate whether each material, system or method listed is relevant to your study. If you are not sure if a list item applies to your research, read the appropriate section before selecting a response.

Materials & experimental systems

n/a	Involved in the study	
☐	☒	Antibodies
☐	☒	Eukaryotic cell lines
☒	☐	Palaeontology and archaeology
☐	☒	Animals and other organisms
☒	☐	Clinical data
☒	☐	Dual use research of concern
☒	☐	Plants

Methods

n/a	Involved in the study	
☒	☐	ChIP-seq
☐	☒	Flow cytometry
☒	☐	MRI-based neuroimaging

Antibodies

Antibodies used

I. Flow Cytometry and FACS sorting
- The following antibodies were purchased by Biolegend and used at a dilution of 1/200: CD45 (clone 30-F11, cat. number 103132, lot number B341465), CD4 (clone GK1.5, cat. number 100402, lot number B326442), CD4 (clone RM4-4, cat. number 116008, lot number B294070), CD4 (clone RM4.4, cat. number 116006, lot number B211830), CD8 (clone 53-6.7, cat. number 100722, lot number B312598), PD-1 (clone 29F.1A12, cat. number 135218, lot number B181687), CD31 (clone MEC13.3, cat. number 102522, lot number B284200), CD90.2 (Thy1.2) (clone 30-H12, cat. number 105328, lot number B259560), CD90.2 (Thy1.2) (clone 53-2.1, cat. number 140304, lot number B253589), PD-L1 (clone 10F.9G2, cat. number 124315, lot number B349076), PD-L1 (clone 10F.9G2, cat. number 124308, lot number B278302), CD11c (clone N418, cat. number 117318, lot number B290152), CD11b (clone M1/70, cat. number 101206, lot number B294051), Ly-6G/Ly-6C (Gr-1) (clone RB8-8C5, cat. number 108408, lot number B220797), I-Ab (clone AF6-120.1, cat. number 116406, lot number B187954), CD80 (clone 16-10A1, cat. number 104707, lot number B295814), CD86 (clone PO3, cat. number 105109, lot number B295021), CD25 (clone PC61, cat. number 102034, lot number B284919), GITR (clone DTA-1, cat. number 126308, lot number B224259), TCR Vβ5.1, 5.2 (clone MR9-4, cat. number 139507, lot number B262645), CD44 (clone IM7, cat. number 103032, lot number B333710), F4/80 (clone BM8, cat. number 123110, lot number B293269), CD69 (clone H1.2F3, cat. number 104507, lot number B169987), CD146 (clone ME-9F1, cat. number 134710, lot number B354104), CD326 (Ep-CAM) (clone G8.8, cat. number 118216, lot number B246071), CD54 (clone YN1/1.7.4, cat. number 116105, lot number B365414).
- The following antibodies were purchased by BD Biosciences and used at a dilution of 1/200: PD-L1 (clone MIH5, cat. number 564716, lot number 0023985), H-2Kb (clone AF6-88.5, cat. number 553569, lot number 10286), CD48 (clone HM48-1, cat. number 740353, lot number 0299172).
- The following antibodies were purchased by Biolegend and used at a dilution of 1/50: Foxp3 (clone 150D, cat. number 320012, lot number B308361), Foxp3 (clone MF014, cat. number 126410, lot number B339204), Ki-67 (clone 16A8, cat. number 652425, lot number B277015), IFN-γ (clone XMG1.2, cat. number 505808, lot number B278613).
- The following antibodies were purchased by ThermoFisher Scientific and used at a dilution of 1/200: TCR Vα2 (clone B20.1, cat. number 12-5812-82), PD-L2 (clone 122, cat. number 11-9972-82, lot number E00936-1635).
- The following antibody was purchased by Abcam and used at a dilution of 1/100: alpha smooth muscle actin (α-SMA) (clone 1A4, cat. number ab8211, lot number GR3287578-8).

II. Immunofluorescence
a. Mus musculus tissue samples - The following antibodies were used:
Foxp3 (company Novus Biologicals, cat. number NB100-39002, lot number J-3, dilution 1:100), FITC α-SMA (company Abcam, clone 1A4, cat. number ab8211, lot number GR3287578-8, dilution 1:100), AF488 CD3 (company Biolegend, clone 17A2, cat. number 100212, dilution 1:20), Alexa Fluor 647 anti-rabbit IgG (company Invitrogen, clone A21245, cat. number A-21246, lot number 2069609, dilution 1:200).
b. Mus musculus cell immunofluorescence - The following antibodies were used:
Lamp-1 (company Santa Cruz Biotechnology, clone 1D4B, cat. number sc-19992, lot number L2313, dilution 1:400), p62 (company MBL, cat. number PM045, lot number 020, dilution 1:500), LC3 (company NanoTools, clone 5F10, cat. number 0231-100/LC3-5F10, lot number 0231S0603, dilution 1:20), Alexa Fluor 555 anti-mouse IgG (company Invitrogen, cat. number A-21425, dilution 1:500), Alexa Fluor 647 anti-rabbit IgG (company Invitrogen, clone A21245, cat. number A-21246, lot number 2069609, dilution 1:200), and Alexa Fluor 488 anti-rat IgG (company Invitrogen, cat. number A-11006, lot number 1887148, dilution 1:250), CD16/32 (company ThermoFisher, clone 93, cat. number 14-0161-86), TCR beta (company ThermoFisher, clone H57-597, cat. number 14-5961-82), CD11a (company ThermoFisher, clone IBL-6/2, cat. number MA5-17949), PD-1 (company ThermoFisher, clone J43, cat. number 11-9985-85).

III. Immunohistochemistry of human cancer paraffin sections - The following antibodies were used:
Foxp3 (company Zytomed, clone SP97, cat. number 506-3970, dilution 1:50), alpha smooth muscle actin (company Dako, clone 1A4, cat. number M0851, dilution 1:200).

Validation

All commercial antibodies are validated by the vendors in experiments using positive and negative samples as well as isotypic

Validation

antibodies. This information is available at the vendors web under the indicated catalogue number of each antibody. In certain experiments FMO controls were used (where indicated). Additionally, blocking antibodies were tested in our lab by monitoring either cell-specific ablation or blocking of their respective ligand by flow cytometry.

More specifically:
The following antibodies, which were provided by Biolegend, have an in-house validated reactivity against mouse and have been quality tested for flow cytometry, according to information provided at the manufacturer's website, under the indicated catalog number of each antibody: CD45 (clone 30-F11, cat. number 103132, lot number B341465), CD4 (clone GK1.5, cat. number 100402, lot number B326442), CD4 (clone RM4-4, cat. number 116008, lot number B294070), CD4 (clone RM4.4, cat. number 116006, lot number B211830), CD8 (clone 53-6.7, cat. number 100722, lot number B312598), PD-1 (clone 29F.1A12, cat. number 135218, lot number B181687), CD31 (clone MEC13.3, cat. number 102522, lot number B284200), CD90.2 (Thy1.2) (clone 30-H12, cat. number 105328, lot number B259560), CD90.2 (Thy1.2) (clone 53-2.1, cat. number 140304, lot number B253589), PD-L1 (clone 10F.9G2, cat. number 124315, lot number B349076), PD-L1 (clone 10F.9G2, cat. number 124308, lot number B278302), CD11c (clone N418, cat. number 117318, lot number B290152), Ly-6G/Ly-6C (Gr-1) (clone RB8-8C5, cat. number 108408, lot number B220797), I-Ab (clone AF6-120.1, cat. number 116406, lot number B187954), CD80 (clone 16-10A1, cat. number 104707, lot number B295814), CD86 (clone PO3, cat. number 105109, lot number B295021), CD25 (clone PC61, cat. number 102034, lot number B284919), GITR (clone DTA-1, cat. number 126308, lot number B224259), TCR Vβ5.1, 5.2 (clone MR9-4, cat. number 139507, lot number B262645), F4/80 (clone BM8, cat. number 123110, lot number B293269), CD69 (clone H1.2F3, cat. number 104507, lot number B169987), CD146 (clone ME-9F1, cat. number 134710, lot number B354104), CD326 (Ep-CAM) (clone G8.8, cat. number 118216, lot number B246071), CD54 (clone YN1/1.7.4, cat. number 116105, lot number B365414), CD3 (clone 17A2, cat. number 100212).
The following antibody, which was provided by Biolegend, has an in-house validated reactivity against mouse, human, cynomolgus and rhesus and has been quality tested for flow cytometry, according to information provided at the manufacturer's website, under the indicated catalog number: CD11b (clone M1/70, cat. number 101206, lot number B294051).
The following antibody, which was provided by Biolegend, has an in-house validated reactivity against mouse and human, and has been quality tested for flow cytometry, according to information provided at the manufacturer's website, under the indicated catalog number: CD44 (clone IM7, cat. number 103032, lot number B333710).
The following antibodies, which were provided by Biolegend, have an in-house validated reactivity against mouse and have been quality tested for intracellular flow cytometry, according to information provided at the manufacturer's website, under the indicated catalog number of each antibody: Foxp3 (clone MF014, cat. number 126410, lot number B339204), Ki-67 (clone 16A8, cat. number 652425, lot number B277015), IFN-γ (clone XMG1.2, cat. number 505808, lot number B278613).
The following antibody, which was provided by Biolegend, has an in-house validated reactivity against mouse, human and rat, and has been quality tested for flow cytometry, according to information provided at the manufacturer's website, under the indicated catalog number: Foxp3 (clone 150D, cat. number 320012, lot number B308361).
The following antibodies, which were provided by BD Biosciences, have a reactivity against mouse: PD-L1 (clone MIH5, cat. number 564716, lot number 0023985), H-2Kb (clone AF6-88.5, cat. number 553569, lot number 10286), CD48 (clone HM48-1, cat. number 740353, lot number 0299172). According to the manufacturer's website, "the specificity is confirmed by using multiple applications that may include a combination of flow cytometry, immunofluorescence, immunohistochemistry or western blot to test a combination of primary cells, cell lines or transfectant models". More information is provided under the indicated catalog number of each antibody.
The following antibody, which was provided by Novus Biologicals, has a reactivity against mouse, according to information provided at the vendor's website and data provided by a verified customer which are shown at the website, under the indicated catalog number: Foxp3 (cat. number NB100-39002, lot number J-3) and has been reported in the literature to work for mouse immunofluorescence experiments (e.g. PMID 36932450).
The following antibody, which was provided by Abcam is predicted to react with mouse, according to information and product publications (e.g. PMID 25858253 for mouse immunofluorescence experiments) provided at the vendor's website, under the indicated catalog number: alpha smooth muscle actin (α-SMA) (clone 1A4, cat. number ab8211, lot number GR3287578-8).
The following antibodies, which were provided by ThermoFisher Scientific, have an in-house validated reactivity against mouse and have been quality tested for flow cytometry, in accordance with the recommendations of the International Working Group for Antibody Validation (vendor's website link provided: https://www.thermofisher.com/gr/en/home/life-science/antibodies/invitrogen-antibody-validation.html), under the indicated catalog number of each antibody: TCR Vα2 (clone B20.1, cat. number 12-5812-82), PD-L2 (clone 122, cat. number 11-9972-82, lot number E00936-1635).
The following antibodies, which were provided by ThermoFisher Scientific, have an in-house validated reactivity against rabbit and have been quality tested for immunofluorence, in accordance with the recommendations of the International Working Group for Antibody Validation (vendor's website link provided: https://www.thermofisher.com/gr/en/home/life-science/antibodies/invitrogen-antibody-validation.html): Alexa Fluor 647 anti-rabbit IgG (clone A21245, cat. number A-21246, lot number 2069609), Alexa Fluor 647 anti-rabbit IgG (clone A21245, cat. number A-21246, lot number 2069609).
The following antibody, which was provided by ThermoFisher Scientific, has an in-house validated reactivity against mouse and has been quality tested for immunofluorence, in accordance with the recommendations of the International Working Group for Antibody Validation (vendor's website link provided: https://www.thermofisher.com/gr/en/home/life-science/antibodies/invitrogen-antibody-validation.html): Alexa Fluor 555 anti-mouse IgG (polyclonal, cat. number A-21425.
The following antibodies, which were provided by ThermoFisher Scientific, have an in-house validated reactivity against mouse (vendor's website link provided: https://www.thermofisher.com/gr/en/home/life-science/antibodies/invitrogen-antibody-validation.html) and have been verified to work in immunofluorescence experiments from published articles provided at the vendor's website under the indicated catalog number: CD16/32 (clone 93, cat. number 14-0161-86), TCR beta (clone H57-597, cat. number 14-5961-82), CD11a (clone IBL-6/2, cat. number MA5-17949, CD11a (clone IBL-6/2, cat. number MA5-17949), PD-1 (clone J43, cat. number 11-9985-85).
The following antibody, which was provided by ThermoFisher Scientific, has an in-house validated reactivity against rat and has been quality tested for immunofluorence, in accordance with the recommendations of the International Working Group for Antibody Validation (vendor's website link provided: https://www.thermofisher.com/gr/en/home/life-science/antibodies/invitrogen-antibody-validation.html): Alexa Fluor 488 anti-rat IgG (polyclonal, cat. number A-11006, lot number 1887148).
The following antibody, which was provided by Santa Cruz Biotechnology, has been validated to work with mouse, rat and human, according to information and product publications (e.g. PMID 28581446 for mouse immunofluorescence experiments) provided at the vendor's website under the indicated catalog number: Lamp-1 (clone 1D4B, cat. number sc-19992, lot number L2313).
The following antibody, which was provided by MBL Life Science, has a reactivity against human, mouse, rat, hamster, bovine and zebrafish and has been reported in the literature to work for immunocytochemistry, according to information and product publications (e.g. PMID 28581446 for mouse immunofluorescence experiments) provided at the vendor's website under the

indicated catalog number: p62 (cat. number PM045, lot number 020).
The following antibody, which was provided by NanoTools, has a reactivity against human, mouse, rat, dog and hamster according to information provided at the vendor's website under the indicated catalog number: LC3 (clone 5F10, cat. number 0231-100/LC3-5F10) and has been reported in the literature to work for mouse immunofluorescence experiments (e.g. PMID 28581446).

Eukaryotic cell lines

Policy information about cell lines and Sex and Gender in Research

Cell line source(s)	The B16.F10 mouse melanoma cell line, Lewis Lung Carcinoma (LLC) cell line and MB49 mouse bladder carcinoma cell line were kindly provided by Dr. Eliopoulos (School of Medicine, University of Athens, Greece).
Authentication	The cell lines were not authenticated but were used at early passages (5-10) and kept their morphology as checked by microscope.
Mycoplasma contamination	Cell lines were tested negative for mycoplasma contamination, tested by PCR.
Commonly misidentified lines (See ICLAC register)	No commonly misidentified lines were used.

Animals and other research organisms

Policy information about studies involving animals; ARRIVE guidelines recommended for reporting animal research, and Sex and Gender in Research

Laboratory animals	- Mus musculus C57BL/6, female or male, aged 8-12 weeks<br>- Mus musculus αSMA-tk (C57BL/6 background), female, aged 8-12 weeks<br>- Mus musculus αSMAcre (C57BL/6 background), female or male, aged 8-12 weeks<br>- Mus musculus αSMA-RFP C57BL/6 background), female, aged 8-12 weeks<br>- Mus musculus Atg5fl/fl (C57BL/6 background), female or male, aged 8-12 weeks<br>- Mus musculus αSMA-tk;RFP (C57BL/6 background), female, aged 8-12 weeks<br>- Mus musculus αSMAcreAtg5fl/fl (C57BL/6 background), female or male, aged 8-12 weeks<br>- Mus musculus Foxp3gfp.KI (C57BL/6 background), female, aged 8-12 weeks<br>- Mus musculus OTII (C57BL/6 background), female or male, aged 8-12 weeks<br>- Mus musculus Rag-/-OTII (C57BL/6 background), female or male, aged 8-12 weeks<br>- Mus musculus PD-1-/- (C57BL/6 background), female, aged 8-12 weeks
Wild animals	The study did not involve wild animals.
Reporting on sex	Sex was not considered on study design. In all experiments, sex-matched mice aged between 8 and 12 weeks were used.
Field-collected samples	The study did not involve field-collected samples.
Ethics oversight	All procedures were in accordance with institutional guidelines and approved by the Institutional Committee of Protocol Evaluation together with the Directorate of Agriculture and Veterinary Policy, Region of Attika, Greece (530216/22 July 2020).

Note that full information on the approval of the study protocol must also be provided in the manuscript.

Plants

Seed stocks	*Report on the source of all seed stocks or other plant material used. If applicable, state the seed stock centre and catalogue number. If plant specimens were collected from the field, describe the collection location, date and sampling procedures.*
Novel plant genotypes	*Describe the methods by which all novel plant genotypes were produced. This includes those generated by transgenic approaches, gene editing, chemical/radiation-based mutagenesis and hybridization. For transgenic lines, describe the transformation method, the number of independent lines analyzed and the generation upon which experiments were performed. For gene-edited lines, describe the editor used, the endogenous sequence targeted for editing, the targeting guide RNA sequence (if applicable) and how the editor was applied.*
Authentication	*Describe any authentication procedures for each seed stock used or novel genotype generated. Describe any experiments used to assess the effect of a mutation and, where applicable, how potential secondary effects (e.g. second site T-DNA insertions, mosiacism, off-target gene editing) were examined.*

Flow Cytometry

Plots

Confirm that:

☒ The axis labels state the marker and fluorochrome used (e.g. CD4-FITC).

☒ The axis scales are clearly visible. Include numbers along axes only for bottom left plot of group (a 'group' is an analysis of identical markers).

☒ All plots are contour plots with outliers or pseudocolor plots.

☒ A numerical value for number of cells or percentage (with statistics) is provided.

Methodology

Sample preparation	Single cell suspensions from mouse lymph nodes and spleens were generated by passing them through a 40µm cell strainer. For tumour cell analysis, melanoma tissues were excised and cut into the smallest possible fragments by using an ophthalmic scissor. The minced tissues were incubated for 45 min at 37°C in RPMI medium containing DNase I (0.25 mg/ml, Sigma) and collagenase D (1 mg/ml, Roche). For analysis of tumour infiltrating lymphocytes (TILs), cell suspensions were prepared by passing through a 40µm cell strainer. For analysis of CAFs, cell suspensions were prepared by passing through a 100µm cell strainer.
Instrument	FACS and sorting was performed with BD ARIA III.
Software	Analysis was performed with FlowJo version 10 and v10.7.2<br>Collection of data was performed BD FACS Diva SOFTWARE v 6.1.3.
Cell population abundance	Purity of FACS-sorted samples was >95%.
Gating strategy	Gating strategy of tumour cell suspensions: Live cells were gated according to size (FSC) and complexity (SSC) and doublets were excluded using plots of FSC-H vs SSC-H and FSC-W vs SSC-W. Cancer associated fibroblasts isolated from αSMA-RFP mice were gated as CD45-CD31-αSMA(RFP)+ cells.<br>Gating strategy of spleen and lymph node cell suspensions: Live cells were gated according to size (FSC) and complexity (SSC) and doublets were excluded using plots of FSC-H vs SSC-H and FSC-W vs SSC-W. T regulatory cells isolated from OTII mice were gated as CD4+CD25+GITR+Vα2+Vβ5.1+ cells. T regulatory cells isolated from Foxp3gfp.KI mice were gated as CD4 +Foxp3(GFP)+ cells.

☐ Tick this box to confirm that a figure exemplifying the gating strategy is provided in the Supplementary Information.
