# Supplementary material for: Immunological synapse formation between T regulatory cells and cancer-associated fibroblasts promotes tumour development
Source: Nat Commun. 2024 Jun 11;15:4988. doi: 10.1038/s41467-024-49282-1 (PMC11167033; doi:10.1038/s41467-024-49282-1)
Supplement: Supplementary file 4 — Description of Additional Supplementary Files [file 41467_2024_49282_MOESM4_ESM.pdf]

Description of Additional Supplementary Files

File Name: Supplementary Movie 1

Description: Representative time-lapse video microscopy showing interaction between CAFs and $CD4^+CD25^+GITR^+V\alpha2^+V\beta5.1^+$ Tregs sorted from OTII mice, in the presence of $OVA_{323\text{-}339}$. Related to Figure 3. 4fps, scale bar: 100 pixels.

File Name: Supplementary Movie 2

Description: Representative time-lapse video microscopy showing interaction between CAFs and $CD4^+CD25^+GITR^+V\alpha2+V\beta5.1^+$ Tregs sorted from OTII mice, in the absence of $OVA^{323\text{-}339}$ (vehicle). Related to Figure 3. 4fps, scale bar: 100 pixels.

File Name: Supplementary Movie 3

Description: Representative video from 3D fluorescent confocal microscopic imaging,

showing interaction between CAFs and $CD4^+CD25^+GITR^+V\alpha2^+V\beta5.1^+$ Tregs sorted from OTII mice, in the presence of $OVA_{323\text{-}339}$. Cells were stained for actin, TCR and PD-1, interactions are observed on filopodia extending from α-SMA^+ CAFs. Related to Figure 3. 30fps, scale bar: 1μm.
